# Short version of the Depression Anxiety Stress Scale-21: is it valid for Brazilian adolescents?

**DOI:** 10.1590/S1679-45082016AO3732

**Published:** 2016

**Authors:** Hítalo Andrade da Silva, Muana Hiandra Pereira dos Passos, Valéria Mayaly Alves de Oliveira, Aline Cabral Palmeira, Ana Carolina Rodarti Pitangui, Rodrigo Cappato de Araújo

**Affiliations:** 1 Universidade de Pernambuco, Petrolina, PE, Brazil.

**Keywords:** Affective symptoms, Surveys and questionnaires, Reproducibility of results, Adolescent, Validation studies, Psychiatric status rating scales

## Abstract

**Objective:**

To evaluate the interday reproducibility, agreement and validity of the construct of short version of the Depression Anxiety Stress Scale-21 applied to adolescents.

**Methods:**

The sample consisted of adolescents of both sexes, aged between 10 and 19 years, who were recruited from schools and sports centers. The validity of the construct was performed by exploratory factor analysis, and reliability was calculated for each construct using the intraclass correlation coefficient, standard error of measurement and the minimum detectable change.

**Results:**

The factor analysis combining the items corresponding to anxiety and stress in a single factor, and depression in a second factor, showed a better match of all 21 items, with higher factor loadings in their respective constructs. The reproducibility values for depression were intraclass correlation coefficient with 0.86, standard error of measurement with 0.80, and minimum detectable change with 2.22; and, for anxiety/stress: intraclass correlation coefficient with 0.82, standard error of measurement with 1.80, and minimum detectable change with 4.99.

**Conclusion:**

The short version of the Depression Anxiety Stress Scale-21 showed excellent values of reliability, and strong internal consistency. The two-factor model with condensation of the constructs anxiety and stress in a single factor was the most acceptable for the adolescent population.

## RESUMO

**Objetivo:**

Avaliar a reprodutibilidade interdias, a concordância e a validade do construto da versão reduzida da *Depression Anxiety Stress Scale-*21 aplicada a adolescentes.

**Método:**

A amostra foi composta por adolescentes de ambos os sexos, com idades entre 10 e 19 anos, recrutados de escolas e centros esportivos. A validade de construto foi realizada por análise fatorial exploratória, e a confiabilidade foi calculada para cada construto, por meio de coeficiente de correlação intraclasse, erro padrão de medida e mudança mínima detectável.

**Resultados:**

A análise fatorial combinando os itens correspondentes a ansiedade e estresse em um único fator, e depressão em um segundo fator apresentou melhor adequação de todos os 21 itens, com cargas fatoriais mais altas em seus respectivos construtos. Os valores de reprodutibilidade para a depressão foram coeficiente de correlação intraclasse com 0,86, erros padrão de medida com 0,80 e mudança mínima detectável com 2,22 e, para a ansiedade/estresse, foram coeficiente de correlação intraclasse com 0,82, erro padrão de medida com 1,80 e mudança mínima detectável com 4,99.

**Conclusão:**

A versão reduzida da *Depression Anxiety Stress Scale-*21 apresentou excelentes valores de confiabilidade e também uma forte consistência interna. O modelo de dois fatores com a condensação dos construtos ansiedade e estresse em um único fator foi o mais aceitável para a população adolescente.

## INTRODUCTION

The short version of the Depression Anxiety Stress Scale-21 (DASS-21) was developed to provide a self-report measure of anxiety, depression and stress signals. During the development process, it was established that the main symptoms of depression are low self-esteem, hopelessness, devaluation of life, self-deprecation and inertia. The main symptom of anxiety is physiological arousal. The stress construct of the scale emerged empirically during the development of the depression and anxiety scales, through aggregation of items relating to difficulty relaxing, tension, impatience, irritability and restlessness.^(1)^

The DASS-21 has been translated and validated in many languages and used with several ethnic groups.^(2-9)^ It is widely used to assess symptoms of mental suffering in clinical and non-clinical adult samples.^(10-15)^ A growing number of studies of adolescents apply the DASS-21 to identify signs of anxiety, depression and stress; however, most studies investigating the validity of the DASS-21 constructs were conducted with adults, making it difficult to extrapolate the scale efficiency items to recognize the physiological symptoms in adolescents.

The psychometric properties of the DASS-21 in population under 18 years of age suggest that the emotional symptoms in this age group are similar to those in adults, but this tool requires further improvement.^(16)^ There have been no construct validation studies of the Portuguese language version of the DASS-21 for adolescents. Therefore, it is necessary an exploratory factor analysis that allows data to speak for themselves, that is, would let the structure that is designed for data suggest the most appropriate factor model independently.^(17)^ Moreover, the scarcity of studies confirming the test-retest reproducibility of DASS-21 decreases the accuracy of the measurement results obtained with the instrument.^(18)^

## OBJECTIVE

To evaluate the interday reproducibility, the agreement and validity of the construct of the short version of the Depression Anxiety Stress Scale in adolescents.

## METHODS

### Sample

The sample consisted of adolescents of both sexes, aged between 10 and 19 years, who were recruited from schools and sports centers in the city of Petrolina (PE), Brazil, in 2015. The minimum sample size required for factor analysis is usually ten subjects per item and, at least, a total of one hundred subjects.^(17)^ To calculate the sample size required for reproducibility analysis, we used the G*Power 3.1.7 software,^(19)^ specifying α=0.05; β=0.10 (90% power); correlation ratio for the null hypothesis (ρH_0_)=0.40, proportion of correlation for alternative hypothesis (ρH_1_)=0.80, and an attrition rate of 20%. The required minimum sample size was estimated at 31 subjects.

Depression Anxiety Stress Scale-21 was administered by trained evaluators to clarify possible doubts of adolescents, without interfering in their answers. The scale was applied after the participants and their legal guardians signing the Informed Consent Form. The adolescents answered the DASS-21 individually in classrooms with the presence only of the evaluator. Thirty-one adolescents were recruited to reevaluation, with a 1-week interval between assessment. Participants who failed to complete the scale were not included in the analysis. The study was approved by the Research Ethics Committee of the *Universidade de Pernambuco* (UPE), under protocol number 944.548, CAAE: 38321114.0.0.0000.5207.

### Instrument

The Brazilian version of the DASS-21 is a validated Portuguese translation^(9)^ of the original scale. It is a self-report instrument consisting of three seven-item subscales, to assess depression, anxiety and stress over the last week. The responses are given on a 4-point Likert scale, ranging from zero if “I strongly disagree” to 3 if “I totally agree”. Overall scores for the three constructs are calculated as the sum of scores for the relevant seven items multiplied by two. Ranges of scores correspond to levels of symptoms, ranging from “normal” to “extremely serious”.^(9)^ The DASS-21 items and corresponding constructs are detailed in table 1.

**Table 1:** Items Depression Anxiety and Stress Scale-21 with their respective constructs

Item	Question	Construct
1	I found it difficult to calm myself	Stress
2	My mouth felt dry	Anxiety
3	I didn't experience any positive feelings	Depression
4	I had difficulty breathing at times (such as wheezing and breathlessness without having made any physical effort)	Anxiety
5	It was hard for me to have the initiatives to do things	Depression
6	I intended to exaggerate when I reacted to situations	Stress
7	I felt shaky (for example, in my hands)	Anxiety
8	I felt I was always nervous	Stress
9	I got worried about situations in which I could have panicked and looked ridiculous	Anxiety
10	I felt I had no desire for anything	Depression
11	I felt restless	Stress
12	I found it difficult to relax	Stress
13	I felt depressed and had no motivation	Depression
14	I was intolerant of the things that kept me from continuing to do what I had been doing	Stress
15	I felt like I was going to panic	Anxiety
16	I didn't feel enthusiastic about anything	Depression
17	I felt like I was worthless as a person	Depression
18	I felt like I was being a little too emotional/sensitive	Stress
19	I knew my heartbeat had changed even though I hadn't done anything physically rigorous (*e.g.* increased heart rate. irregular heartbeat)	Anxiety
20	I felt afraid for no reason	Anxiety
21	I felt there was no meaning to life	Depression

Source: Vignola RC, Tucci AM. Adaptation and validation of the depression, anxiety and stress scale (DASS) to Brazilian Portuguese. J Affect Disord. 2014;155:104-9.^(9)^

### Statistical analysis

Test-retest reproducibility was assessed in terms of intraclass correlation coefficient (ICC) with 95% confidence interval (95% CI); ICC values above 0.75 were interpreted as an indication of excellent reliability, values between 0.40 and 0.74 were taken as an indication of good reliability, and >0.40 as an indication of poor reliability.^(20)^ The standard error of measurement (SEM) was calculated to estimate the change in each score and the minimal detectable change (MDC) was also calculated to determine the minimum clinically significant change. The single-sample *t*-test was used to assess systematic differences between test-retest scores, with the significance level set at p=0.05. Absolute agreement was analyzed by preparing the Bland-Altman plot for the first and second assessment results, based on a scatter plot of the difference of the two evaluations and the average of the two evaluations. This provided a visual representation of biases, errors, limits of agreement, outliers and trends.

Construct validity was analyzed using exploratory factor analysis of principal components with varimax rotation. Taking into account the sample size of 310 participants, the factor loading values >0.40 were considered strong.^(21)^ The internal consistency of the subscale for the three constructs was assessed using Cronbach’s α; values between 0.70 and 0.80 indicated a reliable scale, although values below 0.70 were considered acceptable for physiological constructs.^(22)^ We also calculated individual item-total correlations for each construct. This is a structural measure of the validity of a scale, indicating that a given item measures the construct with which it is associated, rather than the others. An item with good validity is more closely correlated with the total score for the construct with which it is associated than the total scores for other constructs.^(9)^

All statistical analyses were conducted using the software Statistical Package for Social Sciences (SPSS) version 20.0, and Graph PadPrism version 5.03.

## RESULTS

### Sample

The sample consisted of 310 adolescents, 179 (57.7%) males, overall mean age of the sample was 14.16 (±2.12) years. Thirty-one adolescents were recruited for reproducibility analysis, but six participants dropped out; hence the reassessment sample comprised 25 adolescents (14 females), giving a statistical power of 89.64%. The interval between assessments was 1 week, and re-evaluated participants had a median age of 18 years (interquartile range=4).

### Internal consistency and construct validity

The DASS-21 was found to have strong internal consistency. Anxiety Cronbach’s α was 0.80; depression was 0.80; stress was 0.77; overall was 0.88. Cronbach’s α for the combination of the anxiety and stress constructs was 0.82, also indicating strong internal consistency. The item-total correlations for each construct were anxiety with 0.77, depression with 0.78 and stress with 0.80. The correlations between constructs were 0.57 for anxiety and depression, 0.70 for anxiety and stress and 0.60 for depression and stress. The scores for the Kaiser-Meyer-Olkin measure of sampling adequacy (KMO=0.885) and the Bartlett sphericity test (approximate χ^2^ of 2106.950, comp=0.000) confirmed that the data were suitable for factor analysis, and that the adequacy of the model was excellent.^(22)^

In the construct of analysis of DASS-21 for three factors, all items corresponding to the constructs anxiety and depression obtained their highest factor loadings in their respective constructs, except for stress, as shown in table 2. The items 8, 6 and 11 of the stress construct had higher factor loading values in its source construct, since the items 14 and 18, despite having greatest factor loadings for stress, 0.48 and 0.47, respectively, they also appear with high load factor to construct anxiety, with 0.43 and 0.45, respectively. The most problematic items were 1 and 12, which greater load on anxiety, despite being nominally associated with stress. The item-total correlations indicated that all 21 items correlated more strongly with the scores for the construct with which they were associated. The item-total correlations ranged between 0.54 and 0.69 for anxiety, between 0.56 and 0.75 for depression, and between 0.57 and 0.68 for stress.

**Table 2:** Main component matrix with varimax rotation forced to three factors (anxiety, depression and stress) and correlation matrix of depression anxiety and stress, in accord with Depression Anxiety and Stress Scale-21

Item	Factor loading			Correlation		
	
	Anxiety	Depression	Stress	Anxiety	Depression	Stress
A15	0.75			0.57	0.41	0.41
A20	0.69			0.60	0.43	0.49
A07	0.66			0.54	0.23	0.35
A09	0.61			0.61	0.37	0.50
A19	0.52			0.69	0.31	0.46
A04	0.47			0.66	0.28	0.41
A02	0.40			0.54	0.23	0.33
D17		0.76		0.44	0.70	0.38
D21		0.75		0.43	0.75	0.42
D10		0.70		0.30	0.69	0.34
D03		0.63		0.23	0.61	0.26
D05		0.57		0.32	0.56	0.35
D13		0.57		0.54	0.71	0.57
D16		0.51		0.37	0.62	0.36
S08			0.82	0.34	0.26	0.57
S11			0.69	0.43	0.39	0.68
S06			0.58	0.38	0.35	0.60
S14	0.43		0.48	0.45	0.38	0.62
S18	0.45		0.47	0.48	0.40	0.63
S01	0.42			0.49	0.32	0.64
S12	0.44		0.40	0.48	0.40	0.64
% Explained variance (total: 46.45)	17.82	15.70	12.93			

A: anxiety items; D: depression items; S: stress items.

Kaiser-Meyer-Olkin measure of sampling adequacy: 0.885. Bartlett’s test of sphericity (approximate χ^2^): p=0.000 2106.950.

Depression Anxiety and Stress Scale-21 had a three-dimension support proposed by the original author. Nonetheless, considering that the three-factor model resulted in some items with similar or stronger loads on nominally non-core constructs, we also conducted varimax orthogonal analysis for a two-factor model (Table 3), combining the items related to anxiety and stress into a single factor, with the items related to depression contributing to the second factor. A better match for all 21 items with higher factor loadings in their respective constructs was observed. Only item 13 (depression) loaded strongly on both factors and it still loaded most strongly on the depression factor. Once again we calculated item-total correlations; based this time on two factors (anxiety-stress and depression). The item-total correlations were between 0.46 and 0.62 for anxiety-stress items, and between 0.56 and 0.75 for depression items. The model with two factors also presented items with the difference between the correlations lower than 0.20; however, to a lesser extent, it was observed in items 20, 6 and 15 of depression.

**Table 3:** Main component matrix with varimax rotation forced to two factors (anxiety/stress and depression) and correlation matrix of depression anxiety and stress, in accord with Depression Anxiety and Stress Scale-21

Item	Factor Loading		Correlation	
	
	Anxiety/stress	Depression	Anxiety/stress	Depression
S18	0.64		0.60	0.40
S11	0.63		0.61	0.39
S14	0.63		0.59	0.38
A09	0.62		0.60	0.37
A20	0.62		0.59	0.43
A07	0.60		0.47	0.22
S06	0.59		0.54	0.35
S12	0.58		0.61	0.40
A15	0.57		0.53	0.41
S08	0.57		0.50	0.25
A19	0.57		0.62	0.31
S01	0.53		0.61	0.32
A04	0.49		0.57	0.28
A02	0.47		0.46	0.23
D17		0.77	0.44	0.70
D21		0.77	0.46	0.75
D10		0.71	0.35	0.69
D03		0.62	0.27	0.61
D13		0.58	0.36	0.56
D05		0.57	0.60	0.71
D16		0.52	0.39	0.62
% Explained variance (total: 40.44)	30.92	9.52		

A: anxiety items; D: depression items; S: stress items.

Kaiser-Meyer-Olkin measure of sampling adequacy: 0.885; Bartlett’s test of sphericity (approximate χ^2^): p=0.000 2106.950.

### Test-retest reproducibility

**Figure1:**
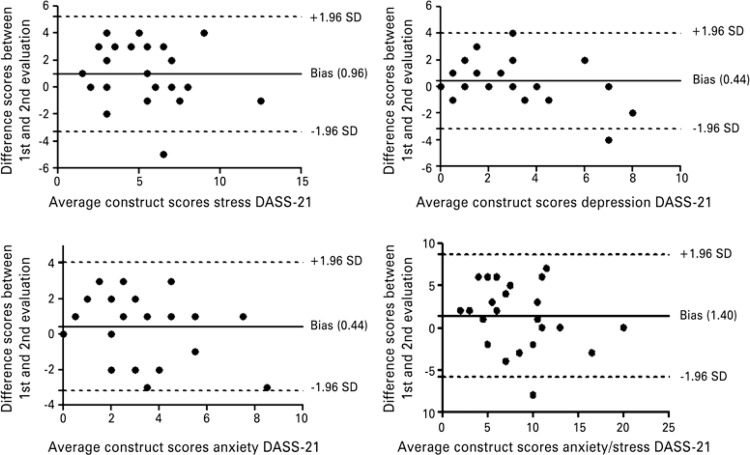
Bland-Altman plots for the different scores in the evaluation and re-evaluation and the mean scores of each individual construct and combination

SD: standard deviation; DASS-21: Depression Anxiety and Stress Scale-21.

The intraclass correlation coefficient values for the DASS-21 indicated excellent reliability, with 95%CI, SEM and MDC for each DASS-21 construct, and are shown on table 4. The agreement analysis for both assessments of each construct is displayed on figure 1. The Student’s *t* test was significant for all constructs, indicating a systematic error type. From the graphs of constructs, one can infer that this error occurred to a lesser extent, considering that the bias of the mean difference was close to zero. It is possible to observe trends in scores in the constructs anxiety and stress, and consequently, the combination scores of both constructs, with a distribution of most individuals above zero, but still within the upper and lower limits of 95%CI. The depression, stress and anxiety-stress scores included some outliers, but, in all cases, they were close to the range limit.

**Table 4:** Values of the intraclass correlation coefficient, confidence interval 95%, standard error of measurement and minimal detectable change to construct the Depression Anxiety and Stress Scale-21

Construct	ICC (CI95%)	SEM	MDC
Anxiety	0.80 (0.54-0.91)	0.88	2.44
Depression	0.86 (0.68-0.94)	0.80	2.22
Stress	0.82 (0.57-0.92)	1.14	3.16
Anxiety/stress	0.82 (0.59-0.92)	1.80	4.99

ICC: intraclass correlation coefficient; SEM: standard error of measurement; MDC: minimal detectable change.

## DISCUSSION

It is important to confirm that a scale is reliable in all populations for which it is used. However, there was only one published evaluation of the test-retest reproducibility of DASS-21, and it did not use the most appropriate statistical tests for this purpose. Bottesi et al.,^(23)^ reported values of r=0.64 for anxiety, r=0.75 for depression and r=0.64 for stress, showing a moderate and positive correlation, but the Pearson’s correlation coefficient effectively measures the relation between the data test-retest, and not the agreement between them. The ICC is considered one of the best measures of validity and, in this study, the ICC values indicated excellent test-retest validity, providing evidence that the DASS-21 is reliable to be used with adolescents.

It is also important to calculate the SEM and the MDC, since they provide further information to assess reproducibility of a test, quantifying the absolute error for scores on the test, and the minimum difference in scores that could not be attributed to measurement error. In this study, the SEM values for DASS-21 scores were small, and the probability of random and systematic error in measurement was also small. Nevertheless, this error was visible when we performed the Student’s *t* test and made the Bland-Altman plots for each construct. The problem most often found in the construct stress was related to the analysis of the combined anxiety-stress construct. The fact that scores were lower on retest may indicate that respondents understood the questions and response scales better having completed the entire questionnaire before. The MDC values were calculated to offer a good reference point for intervention studies. But, likewise other reproducible values, the MDC has not been calculated in the studies that validated the DASS-21.

Considering that the three DASS-21 constructs are based on multiple items, it is appropriate to calculate Cronbach’s α coefficient separately for each construct. In this study, the scale was found to be reliable and the values for internal consistency were comparable with those reported in other recent studies.^(2,4-6,9,16,21,24)^ Cronbach’s α coefficient ranged from 0.74 to 0.86 for anxiety, 0.77 to 0.92 for depression, and 0.70 to 0.90 for stress. Three studies reported Cronbach’s α coefficient for the scale as a whole.^(2,5,24)^ These values and the value observed in this study indicated that the DASS-21 had acceptable internal consistency, no redundant questions and was made up of independent items.^(22)^The Cronbach’s α coefficient showed an increase in the analysis of joint constructs of anxiety and stress, possibly enhancing the role of items that can have more interaction.

In this study, the DASS-21 constructs were strongly and positively correlated. The correlation between anxiety and stress was highest, corroborating other studies.^(2,6,16)^ Other investigations have reported higher correlations between anxiety and depression^(24)^ and between depression and stress^(4,21,25)^ than we found in this study. The strong correlations between constructs is a relevant indication that there are similarities between items associated with the different constructs, which may present issues that may show signs of concomitant anxiety, depression and stress. This cannot fully explain the results for construct analysis based on three factors, as proposed in the original scale.^(26)^

Three-factor model structural analysis reveals problems, with some items loading strongly on more than one construct or loading strongly on nominally unrelated constructs. The items 14 and 18 (stress) also loaded strongly on anxiety. The problem was most serious for item 12 (stress), which loaded most strongly on anxiety. Opposing the three-factor model with its floating items, the two-factor model achieved a better adaptation of the items, but there was a decline in the explained variance, which is a clear indication that the flaws in both models stem from the interpretation of the items they comprise. Our findings suggest that the representativeness of the DASS-21 dimensions remains questionable even in the two-factor model.

The results of this study do not differ from those of earlier studies^(4-6,9)^ with DASS-21, but they still support a structure of three factors, until for presenting a greater explained variance. The study which validated a version of the DASS-21 for use in Brazil^(9)^ corroborated the original three-factor structure. Although several items loaded strongly on more than one factor, the authors only highlighted item 18, which loaded most strongly on the depression factor despite belonging to the stress construct. They offered an explanation based on cultural factors regarding the concept of sensitivity. Oei et al.,^(5)^ endorsed the three-factor model with the caveat that to achieve acceptable model fit, it was necessary to delete three items from the stress construct which loaded more strongly on the depression factor. They nevertheless found that several items loaded strongly on more than one factor. Other studies^(4,6,24)^ supported the three-factor model, but did not have enough data to reinforce this model or to discuss the problem encountered.

The structural problems we found in the three-factor model were also observed in other exploratory factor analyses of the DASS-21 in the adult population,^(2,7,21)^ and in adolescents.^(27)^ These problems are the main reason for advocating further analysis, including the testing of other models which might fit better. The exploratory factor analysis by Apóstolo et al.,^(7)^ revealed flaws in the original model, and the authors proposed an alternative two-factor model, combining anxiety and stress into a single factor; when tested this model proved a better fit to the data.

Most studies that tried to validate the DASS-21 in specific languages, or for specific ethnic groups, used adult samples. There were few attempts to validate the scale for adolescent populations, since this age group is thought to have problems comprehending the scale and differentiating between some of the symptoms it assesses. The five studies^(16,25,27-29)^ that used adolescent samples reached different conclusions about the best model for this population. Some studies^(16,25,28)^ emphasized the high correlations between the constructs, suggesting that they were not empirically distinguishable, especially among adolescents.

Most studies with adolescent samples concluded that a two-factor model is most appropriate in this population, but with reservations. Tully et al.,^(29)^ and Willemsen et al.,^(27)^ defended a two-factor model alternative, keeping the constructs anxiety and depression, and a new construct is formed by the three constructs condensed into a single factor, called “Negative Affect”. This model, before those tested, had the best adjustment. Duffy et al.,^(28)^ also argued that a two-factor model would be more appropriate, but they proposed new factors: a physiological arousal factor comprising four items from the original anxiety construct (2, 4, 7, 19), and a general negativity factor comprising all other items.

Patrick et al.,^(25)^ argued for a one-factor model on the basis of the high inter-construct correlations, suggesting that adolescents cannot differentiate the three dimensions of the original model, and that, in adolescents, the scale only assesses a single dimension. They note that it is not possible to infer, from the available data, whether this single factor is more or less strongly associated with any of the constructs of the adult model. Szabó^(16)^ also reported strong correlations between the constructs, defended the three-factor model, but concluded that although anxiety and depression are similar in both adults and adolescents, stress is questionable in this age group.

It is important to recognize the limitations of this study. The failure to consider the educational background of the adolescents was an important limitation, since relevant data were not gathered. The wide variation in age (10 to 19 years) in our sample means that there was also wide variation in school grade and hence years of education, which may imply differences in comprehension of the questions that make up the scale. Another limitation was that we did not carry out confirmatory factor analysis; however, given the lack of consensus on the factorial structure of the scale, an exploratory factor analysis seemed more appropriate.

Considering the increasing use of the DASS-21 in studies with adolescents, it is necessary to revise some items, especially those belonging to the stress construct, to minimize problems caused by adolescents’ misinterpretation or lack of comprehension of some items. Thus, the DASS-21 can be a tool that clearly and independently identifies the signs of the three psychological states that compose it, in the adolescent population. It is also necessary to conduct further analyses to determine the reliability of the scale in other populations, so as to have an even more consistent discussion of the values found.

## CONCLUSION

The short version of the Depression, Anxiety Stress Scale-21 has excellent reliability and good internal consistency. In the analysis of agreement, it was possible to infer, though small, the presence of systematic error type in all constructs. This emphasizes the importance of knowing the standard error of measurement and the minimal detectable change values if the scale is to be clinically used. Finally, the two-factor model in which the original anxiety and stress constructs are combined into a single factor appears to be more appropriate for the adolescent population. However, considering the psychometric limitations of the two models, both could be used to assess adolescents.
